# Path Planning of Unmanned Autonomous Helicopter Based on Human-Computer Hybrid Augmented Intelligence

**DOI:** 10.1155/2021/6639664

**Published:** 2021-01-13

**Authors:** Zengliang Han, Mou Chen, Tongle Zhou, Zhiqiang Nie, Qingxian Wu

**Affiliations:** ^1^College of Automation Engineering, Nanjing University of Aeronautics and Astronautics, Nanjing 211106, China; ^2^Science and Technology on Electro-optic Control Laboratory, Luoyang Institute of Electro-Optical Equipment of Avic, Luoyang 471000, China

## Abstract

Unmanned autonomous helicopter (UAH) path planning problem is an important component of the UAH mission planning system. The performance of the automatic path planner determines the quality of the UAH flight path. Aiming to produce a high-quality flight path, a path planning system is designed based on human-computer hybrid augmented intelligence framework for the UAH in this paper. Firstly, an improved artificial bee colony (I-ABC) algorithm is proposed based on the dynamic evaluation selection strategy and the complex optimization method. In the I-ABC algorithm, the following way of on-looker bees and the update strategy of nectar source are optimized to accelerate the convergence rate and retain the exploration ability of the population. In addition, a space clipping operation is proposed based on the attention mechanism for constructing a new spatial search area. The search time can be further reduced by the space clipping operation under the path planning result within acceptable changes. Moreover, the entire optimization process and results can be feeded back to the knowledge database by the human-computer hybrid augmented intelligence framework to guide subsequent path planning issues. Finally, the simulation results confirm that a feasible and effective flight path can be quickly generated by the UAH path planning system based on human-computer hybrid augmented intelligence.

## 1. Introduction

In recent years, the developments in automated and unmanned flight technologies have been of high interest to many military organizations throughout the world [1, 2]. Due to their outstanding capability to work in remote or hazardous situations, the unmanned autonomous helicopters (UAHs) have been widely used for many civil and military applications [3]. Path planning is the key to ensure the successful completion of UAH missions [4]. This particular issue is classified as an NP-Hard optimization problem in the high dimension [5]. In the past few years, a variety of methods have been proposed for the path planning problem of UAHs [6, 7]. Traditional methods, such as Voronoi diagram algorithm [8], A∗ algorithm [9], artificial potential field algorithm [10], and rapidly exploring random tree algorithm [11] were proposed to process the path planning problem. However, most of these methods exist the long search times and precociousness issues when the UAH flight path has complex constraints.

Recently, with the development and application of metaheuristic algorithms, more and more swarm intelligence algorithms have been widely applied to the complex optimization problems [12, 13]. As a kind of nature-inspired algorithms, metaheuristic algorithms were originated from imitating biological interactive behaviors or physical phenomena [14], such as the particle swarm optimization (PSO) algorithm [15], genetic algorithm (GA) [16], and differential evolution (DE) algorithm [17]. In recent years, lots of studies have been investigated on improving and modifying the existing metaheuristic algorithms, such as wavelet mutation strategy differential evolution (WMSDE) algorithm [18], multistrategy-based coevolutionary DE (MPPCEDE) algorithm [19], and multiple strategies quantum-inspired differential evolution (MSIQDE) algorithm [20].

Based on the existing research, recently, series of metaheuristic algorithms had been used to solve the UAH path planning problem [21]. In [22], a modification of wolf pack search (WPS) algorithm was presented for path planning of the unmanned aerial vehicle (UAV). Similarly, a grey wolf optimizer (GWO) was applied for path planning problem in the battle field in [23]. In [24], an improved bat algorithm (I-BA) was proposed to process the 3D path planning problems for unmanned combat air vehicles (UCAV). In [25], the multiverse optimizer (MVO) was used for resolving the 2D path planning problem for the UAV. In [26], the discrete particle swarm optimization (PSO) technique was enhanced and used to the path planning problem for surface inspection based on UAV vision.

In 2005, a novel global optimization algorithm based on swarm intelligence called artificial bee colony (ABC) algorithm was proposed by Karaboga [27]. Compared with other intelligent algorithms, the principle of the artificial bee colony (ABC) algorithm makes the algorithm has the advantages of implementation and flexibility. With the continuous in-depth study of the ABC algorithm, it has been widely applied to resolve many engineering application and control problems, such as system identification, task assignment, and path planning. The ABC algorithm has a superior effect in solving path planning problems. However, it is easy to fall into a local optimal solution during the ABC algorithm iteration. To this end, many improved ABC algorithms have been proposed, such as Rosenbrocks rotational direction strategy [28], Boltzmann selection strategy [29], DE-ABC (differential evolution-ABC) [30], PSO-ABC (particle swarm optimization-ABC) [31], and QE-ABC (quantum evolutionary-ABC) [32]. Although those modified ABC algorithms have been widely used in various path planning problems, they were limited by the inherent limitations of the heuristics. Thus, the path planning results need to be further optimized and improved.

Due to the open-ended nature of the path planning problems, no matter how intelligent computers are, they still cannot completely replace humans. Unmanned systems still need human supervision to take advantage of them. Therefore, it is indispensable to utilize human cognitive capabilities or human-like cognitive models into optimization layer to exploit a new path planning system for the UAH [33]. In recent years, with the development of artificial intelligence, researchers have become interested in a new kind of technique called hybrid-augmented intelligence [34]. From the actual situation, “hybrid-augmented intelligence” has been widely used in the fields of industrial development decision-making, online intelligent learning, medical health care, and human-computer codriving [35–37]. The idea of human-computer integration provides a new research approach for the path planning of the UAH. In this paper, the human-computer hybrid augmented intelligence is applied to the path planning system of the UAH. Combining the human intelligent experience to compensate for the shortcomings of the heuristic algorithm, a feasible flight path can be calculated by the improved algorithm. Furthermore, the search time of the flight path can also be further reduced while ensuring that it meets all constraints.

To sum up, the ABC algorithm has great preponderance in solving the multiconstraints optimization problem. Similarly, the concept of hybrid augmented intelligence relies on the characteristics of human-computer integration, enabling the system to handle the complex and difficult problems. To overcome the defects of the traditional ABC algorithm and improve the quality of the flight path, a path planning system is proposed based on human-computer hybrid augmented intelligence framework for the UAH in this paper. The innovations in this paper are summarized as follows.

- A new path planning system based on human-computer hybrid augmented intelligence framework for UAH is designed to improve the rapidity and optimality of the automatic path planner. The used human-computer hybrid augmented intelligence framework combines the advantages of human and computer for planning a high-quality flight path
- An I-ABC algorithm is proposed based on the dynamic evaluation selection strategy and the complex optimization method for accelerating the convergence rate and maintaining the exploration ability of the population
- A space clipping method is designed based on the attention mechanism for reconstructing the spatial search area. The space clipping operation further reduces the subsequent search time of the flight path for the path planning system
- The simulation studies are executed to comprehensively prove the effectiveness of path planning system based on human-computer hybrid augmented intelligence framework for UAH by various air combat environment models

The remainder of the paper is organized as follows.  Section 2 demonstrates the problem statement.  Section 3 investigates the framework of human-computer hybrid augmented intelligence for path planning.  Section 4 investigates an improved ABC algorithm.  Section 5 investigates the space clipping method based on human optimizing attention mechanism.  Section 6 provides the feasibility and effectiveness of the system by simulation experiment. Finally, the concluding remarks are involved in Section 7.

## 2. Problem Statement

**Figure 1:**
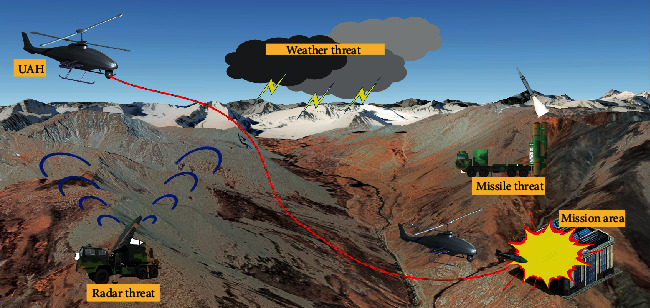
Schematic of UAH path planning mission.

As shown in Figure 1, the path planning is the critical part of the UAH mission assignment system, and it is used to find the optimal flight path under the constraints such as weather threat, terrain threat, radars threat, and missiles threat. In this paper, we mainly study the 2D path planning of the UAH which execute the mission at the same flight altitude. We assume that the UAH maintains constant flight speed and altitude during its mission.

### 2.1. Modeling of UAH Path Planning

In this paper, the starting point and the terminal point are defined as *S* and *T*.

**Figure 2:**
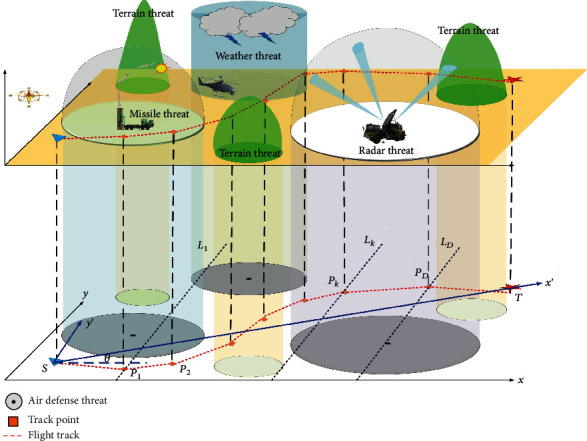
Schematic of 2D UAH battlefield model.

According to the method given in [38] which is shown in Figure 2, we divided *ST* into (*D* + 1) equal parts by *D* vertical lines {*L*_*k*_, *k* = 1, 2, ⋯, *D*}, which intersect *ST* at each segment point. A flight path is formed by connecting the series of points on the new axis. To simplify the calculations, the threat regions covered in this paper are assumed to be circular projections. Therefore, the path planning problem can be converted to the route point coordinate optimization problem.

To simplify the calculation process, a new coordinate frame need to be established. In this section, the segment *ST* is considered as the new *x*-axis [39]. Each path point on the original combat field gets transferred in the new axes as defined in (1).

(1) $$\begin{array}{c} \left(\begin{array}{c} x^{\prime }\\ y^{\prime } \end{array}\right)=\left(\begin{array}{cc} \cos\theta & \sin\theta \\ -\sin\theta & \cos\theta \end{array}\right), \end{array}$$

where *θ* represents the angle between the *x*-axis of the inertial frame and the *x*′ of the body-fixed frame direction, (*x*_*s*_, *y*_*s*_) correspond to the start point on the body-fixed frame.

The damage probability is determined by the distance between the UAH and the threat center. Assuming that the new coordinates of the threat center point are (*x*_*i*_, *y*_*i*_) and the threat radius are *r*_*i*_, then the threat area can be expressed as [40]

(2) $$\begin{array}{c} {\left(x-x_{i}\right)}^{2}+{\left(y-y_{i}\right)}^{2}=r_{i}^{2}. \end{array}$$

### 2.2. Cost Function and Performance Constraints

In this paper, the performance evaluation index of the UAH flight path is composed of the threat cost *J*_threat_^*i*^ and the fuel cost *J*_fule_^*i*^. The total cost and the each cost are described as follows [41].

(3) $$\begin{array}{c} J=\overset{D+1}{\underset{i=1}{\sum }}\left[\tau J_{\text{threat}}^{i}+\left(1-\tau \right)J_{\text{fule}}^{i}\right], \end{array}$$

where *τ* is the weighting parameter between 0 and 1.

**Figure 3:**
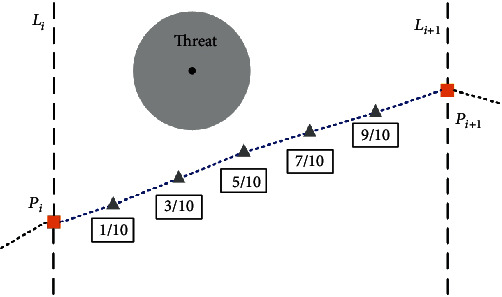
Schematic diagram of flight cost computation.

The flight cost from the point along *L*_*i*_ to the one along *L*_*i*+1_ is calculated at five points (as shown in Figure 3). If the flight path shown above falls into a threat region, the *J*_threat_^*i*^ is calculated as follows.

(4) $$\begin{array}{c} J_{\text{threat}}^{L_{i}\to L_{i+1}}=\frac{l_{i}}{5}\cdot \overset{N_{t}}{\underset{k=1}{\sum }}\left[s_{k}\cdot \left(\frac{1}{d_{0.1,i}^{k}}+\frac{1}{d_{0.3,i}^{k}}+\frac{1}{d_{0.5,i}^{k}}+\frac{1}{d_{0.7,i}^{k}}+\frac{1}{d_{0.9,i}^{k}}\right)\right], \end{array}$$

where *N*_*t*_ is the total number of threats, *l*_*i*_ is the length of the *i*th subtrack, *d*_0.1,*i*_^*k*^ stands for the distance between the 1/10 point on the path and the *k*th threat center, and *s*_*k*_ is regarded as the grade of the *k*th threat.

Assume that the UAH is moving at a constant speed, the *J*_fule_^*i*^ is calculated as follows.

(5) $$\begin{array}{c} J_{\text{fule}}^{i}=\overset{D}{\underset{i=1}{\sum }}\frac{l_{i}}{v}\eta , \end{array}$$

where *l*_*i*_ is the length of the *i*th subtrack, *v* is the speed of the UAH, and *η* is the fuel consumption per unit time of the UAH.

Considering the actual flight situation for the UAH, the yawing angle constraints are introduced as follows.

(6) $$\begin{array}{c} \varphi_{j}=\left|\arctan\left(\frac{y_{j+1}-y_{j}}{x_{j+1}-x_{j}}\right)\right|\leq \varphi^{\max}, \end{array}$$

where *φ*_*j*_ is the yaw angle of the *j*th node and *φ*^max^ is the maximum yawing angle.

## 3. Human-Computer Hybrid Augmented Intelligence Framework for the Path Planning

Introducing human intelligence to the loop of the path planning system can can tackle the fuzzy and uncertain problems [42]. Hence, human and computer cooperate with each other to form a double-sided information exchange and control. The ′1 + 1 > 2′ hybrid augmented intelligence can be realized by conforming human cognitive ability, computer computing, and storage capacities [43].

**Figure 4:**
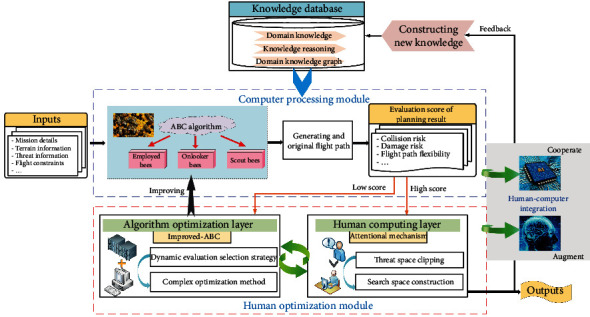
Framework of the human-computer hybrid augmented intelligence.

In this paper, a path planning system based on the human-computer hybrid augmented intelligence framework is designed. The new system integrates the artificial bee colony (ABC) algorithm and human intelligence. Through the human-computer cooperation, the intelligent process ability of the path planning system will be enhanced, and the flight path of the UAH will be efficiently improved. The framework of the path planning system based on the human-computer hybrid augmented intelligence is shown in Figure 4.

The core of the path planning system for the UAH is mainly composed of the following two parts.

### 3.1. Computer Processing Module

When the new combat mission is entered, the computer will calculate an original flight path by the ABC algorithm according to the relevant constraints [44]. This process will always be guided by the experience of the expert knowledge database [45]. The original flight path needs to be evaluated to determined if it becomes the final result. However, it is initially difficult for the traditional ABC algorithm to obtain a high confidence flight path for the UAH path planning system.

### 3.2. Human Optimizing Module

When the original flight path cannot meet the needs of the actual combat, the human-computer hybrid augmented intelligence system will decide whether the result needs human adjustment or human intervention [46]. In this paper, the human optimization module is mainly divided into the following two parts.

- Algorithm optimization layer

The ABC algorithm has a good effect in solving path planning problems; however, it is easy to fall into a local optimal solution during algorithm iteration. In the algorithm optimization layer, the traditional ABC algorithm is improved by the dynamic evaluation selection strategy and the complex optimization method. The improved ABC (I-ABC) algorithm raises the optimisation efficiency, avoids the algorithmic precocity, and plans a high confidence flight path according to the actual task requirements.

(ii) Human computing layer

The path planning based on the I-ABC algorithm can basically meet the mission demands. However, the time of the flight path planning can still be further reduced to improve the efficiency of the planning system. In the human computing layer, the efficiency of the planning system can be further improved by space clipping operation based on the attention mechanism. This step can be used as empirical knowledge to feed back into the expert knowledge base to guide and monitor the entire path planning system, thus enabling human-computer hybrid-augmented features.

## 4. UAH Path Planning Based on I-ABC Algorithm

The ABC algorithm is the most important component of the path planning system for the UAH. The rationality and efficiency of the ABC algorithm in dealing with complex problems will directly affect the results of the path planning system for the UAH. Although the ABC algorithm is widely used at present, it still exposes many problems when it faces complex multiconstraints optimization problems.

In this section, the traditional ABC algorithm will be improved in the algorithm optimization layer to optimize the path planning of the UHA. In the algorithm optimization layer, there are mainly two optimization measures as follows.

- First, in order to prevent the ABC algorithm premature convergence problem, a dynamic evaluation selection strategy is proposed to optimize the follow way of on-looker bees for improving the searching efficiency
- Second, in order to improve the quality of the flight path, the complex method is used to guide the optimization of the swarm. The nectar source is updated during each iteration, the search efficiency of the nectar source is improved, and the algorithm convergence is accelerated

The specific process of the I-ABC algorithm is as follows.

### 4.1. Initialization

There are three kinds of bees, i.e., employed bees, onlooker bees, and scout bees in the ABC algorithm [27]. Accordingly, the triple search capability of the ABC includes of three search phases: employed bee stage, onlooker stage, and scout stage [28]. The position of the nectar source (initial 2D track) is represented by a *D* × *NP*-dimensional matrix *E* = {*e*_*ij*_}, the vector in the *i*th row of the matrix is represented as

(7) $$\begin{array}{c} X_{i}=\left(x_{i1},\cdots ,x_{ij},\cdots ,x_{iD}\right), \end{array}$$

where 1 ≤ *i* ≤ *N*_*p*_, 2 ≤ *j* ≤ (*D* − 1), and *x*_*i*1_ = *x*_*iD*_ = 0.

All the *NP* employed bees need to be randomly initialized by equation (8) [27]. In other words, each track point is randomly generated within a specified range in the feasible solution space.

(8) $$\begin{array}{c} x_{ij}=x_{i}^{\min}+\mathrm{rand}\;\left(0,1\right)\cdot \left(x_{i}^{\max}-x_{i}^{\min}\right), \end{array}$$

where *x*_*i*_^min^ and *x*_*i*_^max^ are the constraints of the *i*th parameter, rand(0, 1) is a random number range from 0 to 1.

Then, the corresponding fitness is calculated as

(9) $$\begin{array}{c} \text{fitness}\left(i\right)=\left\{\begin{array}{cc} \frac{1}{\left[1+obj\left(i\right)\right]}, & \text{obj}\left(i\right)\geq 0,\\ 1+\left|obj\left(i\right)\right|, & \text{obj}\left(i\right)<0, \end{array}\right. \end{array}$$

where obj(*i*) represents the objective function value with respect to *X*_*i*_.

### 4.2. Employed Bee Phase

Each employed bee is related with only one nectar source. The employed bee finds a new candidate solution through changing the nectar source position in its memory based on the local information. In each iteration cycle, the employed bee adopts (10) to search for the better nectar source *V*_*ij*_ around the current nectar source [27].

(10) $$\begin{array}{c} v_{ij}=x_{ij}+\mathrm{rand}\left(1,-1\right)\cdot \left(x_{ij}-x_{kj}\right), \end{array}$$

where *i* = 1, 2, ⋯, *NP*,  *k* ∈ {1, 2, ⋯, *NP*},  *j* ∈ {1, 2, ⋯, *D*}, *k* is a random integer different from *i*, *j* is a random integer, and rand(1, −1) is a random number range from −1 to 1.

During the neighborhood search process, when the location of the nectar source searched by the employed bees exceeds the search boundary constraint, the bee is selected according to (11).

(11) $$\begin{array}{c} x_{ij}=\left\{\begin{array}{cc} x_{j}^{\min}+\partial \left(x_{j}^{\max}-x_{j}^{\min}\right), & x_{ij}\leq x_{j}^{\min},\\ x_{j}^{\min}-\partial \left(x_{j}^{\max}-x_{j}^{\min}\right), & x_{ij}>x_{j}^{\min}, \end{array}\right.\\ \partial \in \mathrm{rand}\left(0,1\right) \end{array}$$

where *x*_*j*_^min^ and *x*_*j*_^max^ are the constraints of the *j*th parameter.

### 4.3. Onlooker Bee Phase

In the onlooker bee phase, the amounts and positions of their nectar sources will be transmitted by the employed bees to onlooker bees. Then, the onlooker bee evaluates all the information of nectar sources transmitted by employed bees, and chooses one nectar source site according to the probability value *p*_*i*_ formulated as [27]

(12) $$\begin{array}{c} p_{i}=\frac{\text{fitness}\left(i\right)}{\sum_{j=1}^{NP}\text{fitness}\left(i\right)}, \end{array}$$

where *NP* represents the number of bees.

However, the traditional following selection method overly focus on the development of excellent nectar sources. Ignoring other potential nectar sources will cause the algorithm's global search capability to decline and reduce the algorithms solution efficiency. For solving this problem, we propose a dynamic evaluation selection strategy instead of the traditional method to follow the location of the nectar sources.

1. Defining the nectar source dynamic evaluation integral value

In this section, the dynamic evaluation integral values of nectar sources are defined as Ψ_1_(*i*) and Ψ_2_(*i*). When the nectar source *i* maintains unchanged, Ψ_1_(*i*) is calculated according to equation (13), and Ψ_2_(*i*) is 0. When the nectar source position *i* is replaced by a better one, Ψ_2_(*i*) is calculated according to equation (14), and Ψ_1_(*i*) is 0.

(13) $$\begin{array}{c} \Psi_{1}\left(i\right)=\left\{\begin{array}{cc} \Psi_{1}\left(i\right)+\text{step}, & \Psi_{1}\left(i\right)<\text{limi}{\text{t}}_{d},\\ \text{limi}{\text{t}}_{d}, & \Psi_{1}\left(i\right)\geq \text{limi}{\text{t}}_{d}, \end{array}\right. \end{array}$$

(14) $$\begin{array}{c} \Psi_{2}\left(i\right)=\left\{\begin{array}{cc} \Psi_{2}\left(i\right)+\text{step}, & \Psi_{2}\left(i\right)<\text{limi}{\text{t}}_{d},\\ \text{limi}{\text{t}}_{d}, & \Psi_{2}\left(i\right)\geq \text{limi}{\text{t}}_{d}, \end{array}\right. \end{array}$$

where Ψ_1_(*i*) is the number of searching near the nectar source *i* but the position has not changed, Ψ_2_(*i*) is the number of searching near the nectar source *i* and the position has changed, and limit_*d*_ is the dynamic update limit parameter, step is the search unit length.

(2) Building the dynamic evaluation function

Considering that there will be more greater possibility of finding excellent nectar sources near the continuously optimized nectar sources. The dynamic evaluation function *F*(*i*) is constructed according to the dynamic evaluation criteria which is written as

(15) $$\begin{array}{c} F\left(i\right)=\left\{\begin{array}{cc} \zeta \left(1-\frac{\Psi_{1}\left(i\right)}{\text{limi}{\text{t}}_{d}}\right), & \Psi_{1}\left(i\right)\neq 0,\\ \zeta \left(1+\frac{\Psi_{2}\left(i\right)}{\text{limi}{\text{t}}_{d}}\right), & \Psi_{1}\left(i\right)=0, \end{array}\right. \end{array}$$

where *ζ* is the base score of the nectar source.

- When Ψ_1_(*i*) = 0, this nectar source may be trapped in a local optimum. Onlooker bees should try to avoid selecting this type of nectar source for further development
- When Ψ_1_(*i*) ≠ 0, this nectar source has been continuously optimized multiple times. Onlooker bees need to try to select this type of nectar source for neighborhood search

The evaluation integral value of the nectar source is calculated by the evaluation function *F*(*i*). The basic score of the nectar source is *ζ*, and the selection possibility of all individuals is guaranteed. The scores of nectar sources with continuously changing positions are more than *ζ*, which has a greater possibility of being followed. The optimized selection probability is calculated by (16).

(16) $$\begin{array}{c} p\prime_{i}=\frac{F\left(i\right)}{\max\left(F\left(i\right)\right)}. \end{array}$$

### 4.4. Scout Bee Phase

In each iteration cycle, the exhausted nectar source will be checked by ABC algorithm after all the employed bees and onlookers complete their searches. If the new position *X*_*i*_ is not improved continuously for a certain time, then the corresponding nectar source will be abandoned by the employed bee. At this time, this employed bee will become a scout bee. According to (10), a new nectar source *v*_*ij*_ will be generated by the scout bee.

In order to improve the efficiency of the nectar source and the quality of the flight path, a complex optimization method is used to guide the swarm. The complex optimization method is a direct search algorithm for finding constrained optimization problems [47]. In solving nonlinear problems, by virtue of its freedom from the constraints of the research problem and the objective function, it has a wide range of applicability and can be embedded in many other algorithms to guide the problem to an optimal or suboptimal solution.

**Figure 5:**
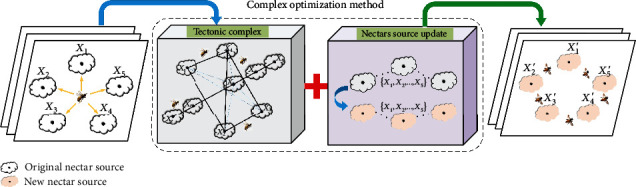
Schematic model of the complex method for five nectar sources.

From the nectar sources before the end of each iteration, *u* nectars are selected to construct the complex geometry, which is ordered as (*X*_1_, *X*_2_, ⋯*X*_*u*_) according to the size of the objective function value from best to worst (as shown in Figure 5). The process of the complex optimization method is as follows.

**Step 1 .**

Centroid calculation

(17) $$\begin{array}{c} X_{c}=\frac{1}{u-1}\overset{u-1}{\underset{i=1}{\sum }}X_{i}. \end{array}$$

**Step 2 .**

Reflection point calculation

(18) $$\begin{array}{c} X_{r}=X_{c}+\alpha \left(X_{c}-X_{u}\right), \end{array}$$

where *α* ∈ (0, 1) is the reflection coefficient. If *X*_*r*_ is better than *X*_*u*_, *X*_*u*_ will be replaced by *X*_*r*_; otherwise, go to Step 4.

**Step 3 .**

Extension point calculation

(19) $$\begin{array}{c} X_{e}=X_{r}+\beta \left(X_{r}-X_{c}\right), \end{array}$$

where *β* ∈ (0, 1) is the extension coefficient. If *X*_*e*_ is better than *X*_*u*_, *X*_*u*_ will be replaced by *X*_*e*_; otherwise, go to Step 4.

**Step 4 .**

Systolic point calculation

(20) $$\begin{array}{c} X_{s}=X_{u}+\chi \left(X_{c}-X_{u}\right), \end{array}$$

where *χ* ∈ (0, 1) is the systolic coefficient. If *X*_*s*_ is better than *X*_*u*_, *X*_*u*_ will be replaced by *X*_*s*_; otherwise, go to Step 1.

In this paper, the purpose of the complex optimization method is to emphasize the “replacement” effect, not the traditional “search for excellence.” So when the convergence termination condition is setted, it is only necessary for the algorithm to reach the required number of iterations, which is related to the number of selected nectar sources. The convergence termination is as follows.

(21) $$\begin{array}{c} N_{\text{complex}}=u. \end{array}$$

**Figure 6:**
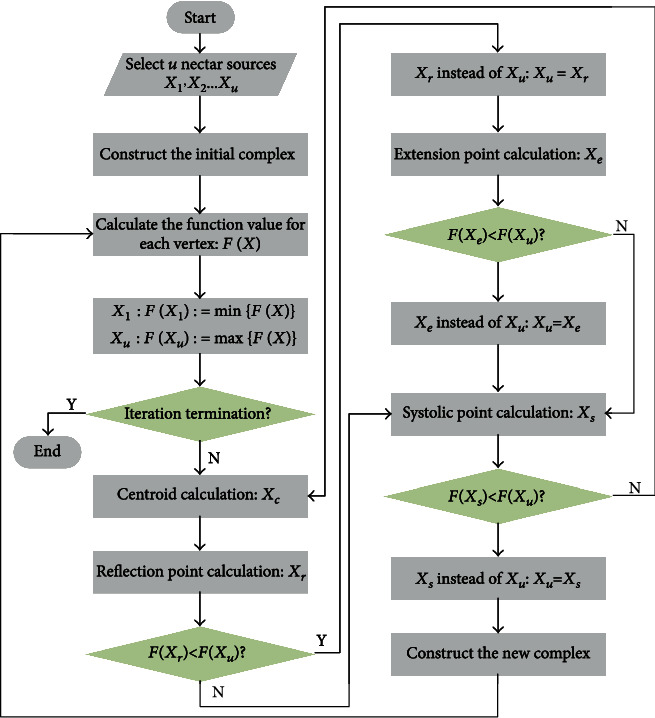
Flow diagram of the complex optimization method.

The flow diagram of the complex optimization method is shown in Figure 6.

By the complex optimization method, the original *u* worst nectar sources in the search space are replaced. The complex geometry keeps reflecting, extending, and contracting in the above ways to make the nectar source approach the optimal location. Since the nectar source is updated during each iteration, the problem of dimensionality reduction due to failure to take into account the correlation and number of points of the initial complex is avoided.

**Figure 7:**
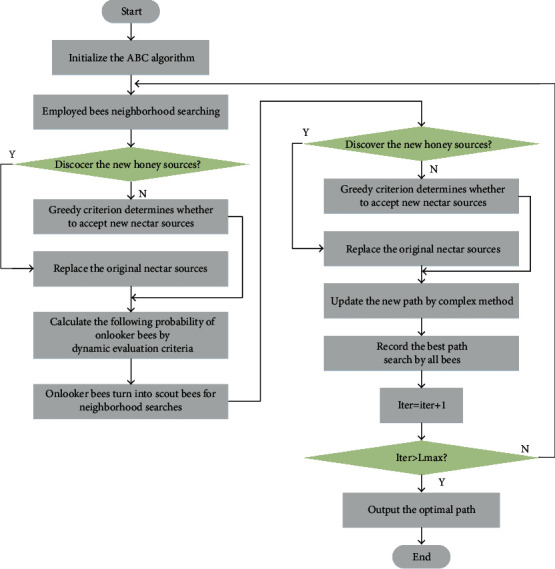
Flow diagram of the I-ABC.

To overcome the defects exposed by the traditional ABC algorithm in dealing with the path planning of the UAH, an I-ABC algorithm is proposed based on the dynamic evaluation selection strategy and complex optimization method in the algorithm optimization layer. The I-ABC algorithm avoids the premature convergence problem, reduces the algorithm search time, and improves the quality of the flight path. The flow diagram of the I-ABC algorithm is shown in Figure 7.

During the iteration, if an employed bee searches globally but finds no better nectar source, or if an onlooker bee searches around an employed bee and finds no better nectar source, the invalid trail time trial plus one. On the other hand, when any better nectar source can be searched by the *i*th employed bee, the relevant trial(*i*) is set to zero immediately. At the end of each iteration, it is necessary to determine whether any trial(*i*) outpaces a certain threshold Limit. If trial(*i*) > Limit, the *i*th employed bee will be diametrically replaced by a scout bee. A scout bee still uses (8) to point to a randomly initialized location in the food source. The pseudocode of the I-ABC algorithm are shown in Algorithm 1.

**Remark 1 .**

The I-ABC algorithm can be divided into two phases. As the first phase of the program, the initialization phase is executed one time at the start, and the other phases are executed in each cycle. The computational complexity is mostly affected by the phase of the algorithm.

The computation is applied to the population with size of *N*, the individuals' position in population is a vector with size of *D*, and *O* is the asymptotic time complexity. The computing complexity *T*(*n*) of each phase is shown as follows:

- Initialization phase

(22) $$\begin{array}{c} T_{1}\left(n\right)=\max\left[O\left(1\right),O\left(N\cdot D\right)\right]=O\left(N\cdot D\right). \end{array}$$

(ii) Optimization phase

(23) $$\begin{array}{c} T_{2}\left(n\right)=\max\left[O\left(N^{2}\cdot D\right),O\left(N\cdot D\right),O\left(N\cdot D\right),O\left(N\cdot D\right)\right]=O\left(N^{2}\cdot D\right). \end{array}$$

Therefore, the maximum computing complexity of the I-ABC algorithm is shown as

(24) $$\begin{array}{c} T\left(n\right)=T_{1}\left(n\right)+T_{2}\left(n\right)=\max\left[O\left(N\cdot D\right),O\left(N^{2}\cdot D\right)\right]=O\left(N^{2}\cdot D\right). \end{array}$$

So it proves that this algorithm owns fast execution speed.

## 5. Space Clipping Operation Based on Attentional Mechanism

**Figure 8:**
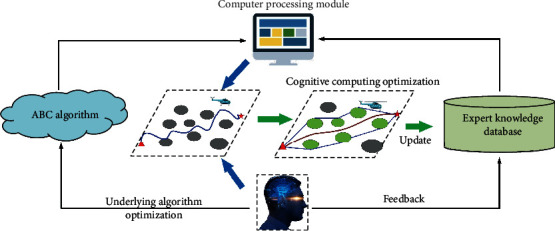
Diagram of human-computer collaboration.

As shown in Figure 8, the incorporation of human intelligence is an important feature of the human-computer hybrid augmented intelligence framework. In order to further reduce the flight path search time and improve the efficiency of the UAH path planning system, a human computing layer is designed for the refinement operation of the flight path. Combined with the prior knowledge and human intelligence, the UAH flight space will be clipped by the spatial attention mechanism.

### 5.1. Attentional Mechanism

**Figure 9:**
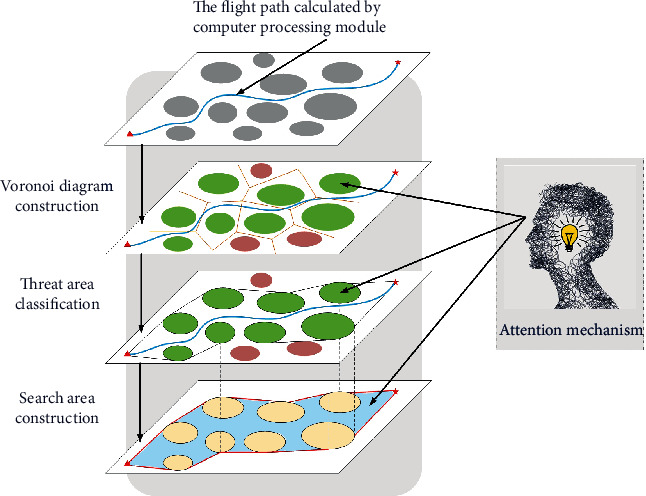
Space clipping operation based on attentional mechanism.

The main idea of the human computing layer presented in this paper is the space clipping based on the attention mechanism. The attention mechanism is a brain signal processing mechanism that is unique to humans [48]. The flight space clipping operation mimics the process by which the human brain rapidly sifts high-value information from large amounts of data through limited attentional resources [49]. The principle of the mechanism is shown in Figure 9.

The division of the threat areas will be determined by the degree of association between the flight path and the threat. Eventually, the spatial area of the path planing will be redrawn.

### 5.2. Space Clipping Operation

In order to meet the constraint of minimizing the length of the path, the flight path can only through the safe space between a part of the threat areas. Numerous studies show that the unrelated threats will increase the search time of the swarm intelligence algorithm and reduce the efficiency of the path planning system. Therefore, it is crucial to distinguish different threat areas by the human computing layer. Combining the priori path planning results knowledge, the UAH flight space can be clipped by following steps.

1. Voronoi diagram construction

The Voronoi diagram is widely used in terrain processing and other areas of division [8]. The points on each edge of the Voronoi diagram polygon are equidistant from the corresponding two threat points. In other words, all points on the edge of the Voronoi diagram are as far away as possible from the threat.

**Step 5 .**

**Figure 10:**
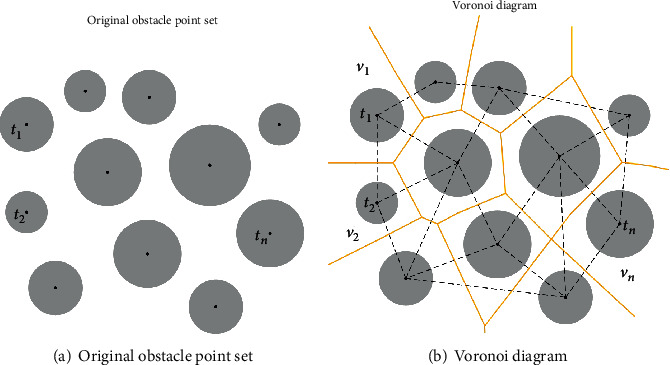
Process of the space clipping operation.

As shown in Figure 10(a), the threat region is considered as a point *t*_*n*_, and all threat regions form a scatter set *T* of finite distances, that is, *T* = {*t*_1_, *t*_2_, ⋯, *t*_*n*_}.

**Step 6 .**

As shown in Figure 10(b), construct Delaunay triangles by connecting discrete points *t*_*n*_ into triangles. Find the edges of the Voronoi diagram by traversing the triangle chain table and draw the Voronoi diagram based on the final result.

The Voronoi diagram divides the flight path planning space into *n* convex polygons *v*_*i*_(*i* = 1, 2, ⋯, *n*) centered on the threat area, and the Voronoi diagram composed of *V* = {*v*_1_, *v*_2_, ⋯, *v*_*n*_} satisfies the following two conditions.

1. Each convex polygon includes one and only one threat area, that is, ∀*v*_*i*_ ∈ *V*, ∃!*t*_*j*_ ∈ *T*, where *t*_*j*_ ∈ *v*_*i*_, ∀*t*_*k*_ ∈ *T*(*k* ≠ *j*), *t*_*k*_ ∉ *v*_*j*_.
2. Suppose that *d*(*x*, *y*) is the Euclid distance on ℝ^2^, if *t*_*j*_ ∈ *v*_*i*_, so that ∀*t* ∈ *v*_*i*_, ∀*t*_*k*_ ≠ *t*_*j*_, where *d*(*x*, *x*_*j*_) ≤ *d*(*x*, *x*_*k*_), *i*, *j* = 1, 2 ⋯ , *n*.
3. Threat areas classification

Assume that the flight path is *C*_path_, the edge between threat *t*_*i*_ and threat *t*_*j*_ is *L*_*v*_*i*,*j*__, the intersection of the flight path and the edge of the convex polygon is *Ξ*_*i*,*j*_^*k*^, where *i* ≠ *j* and *i*, *j* = 1, 2, ⋯, *n*, and *k* is the number of the intersection. The guidelines for determining the type of threat areas are as follows.

- If *C*_path_ intersects *L*_*v*_*i*,*j*__ and the intersection point is *Ξ*_*i*,*j*_^*k*^ (*k* ≥ 1), threat *t*_*i*_ and threat *t*_*j*_ are defined as associated threats. When the position of *Ξ* is located at the intersection of the three edges of the convex polygon, *Ξ* = *Ξ*_*h*,*i*,*j*_^*k*^(*h* ≠ *i* ≠ *j*, *h*, *i*, *j* = 1, 2, ⋯, *n*, *k* = 1); threat *t*_*h*_, threat *t*_*i*_, and threat *t*_*j*_ are defined as associated threats
- If there is no intersection between *C*_path_ and *L*_*v*_*ij*__, that is, *Ξ*_*ij*_^*k*^ does not exist, threat *t*_*i*_ and threat *t*_*j*_ are defined as unrelated threats

**Figure 11:**
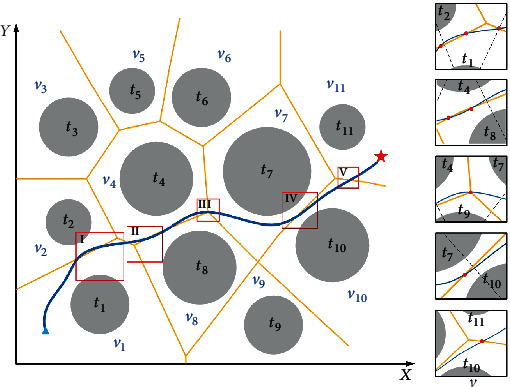
The distribution of *Ξ*_*i*,*j*_^*k*.^.

As shown in Figure 11, the flight space includes 11 threat areas (*T* = {*t*_1_, *t*_2_, ⋯, *t*_11_}), and the Voronoi diagram divides them into 11 convex polygons (*V* = {*v*_1_, *v*_2_, ⋯, *v*_11_}). According to the above theory, the threat areas in Figure 11 can be judged as follows.

- Part I

*C*_path_ and *L*_*v*_1,2__ intersect at *Ξ*_1,2_^1^ and *Ξ*_1,2_^2^; threat *t*_1_ and threat *t*_2_ are associated threats; *C*_path_ and *L*_*v*_1,4__ intersect at *Ξ*_1,4_^1^; threat *t*_1_ and threat *t*_4_ are associated threats.

(ii) Part II

*C*_path_ and *L*_*v*_4,8__ intersect at *Ξ*_4,8_^1^ and *Ξ*_4,8_^2^; threat *t*_4_ and threat *t*_8_ are associated threats.

(iii) Part III

*C*_path_ and *L*_*v*_4,7__ and *L*_*v*_4,8__ and *L*_*v*_7,8__ at *Ξ*_4,7,8_^1^; threat *t*_4_, threat *t*_7_, and threat *t*_8_ are associated threats.

(iv) Part IV

*C*_path_ and *L*_*v*_7,10__ intersect at *Ξ*_7,10_^1^; threat *t*_7_ and threat *t*_10_ are associated threats.

(v) Part V

*C*_path_ and *L*_*v*_10,11__ intersect at *Ξ*_10,11_^1^; threat *t*_10_ and threat *t*_11_ are associated threats.

**Figure 12:**
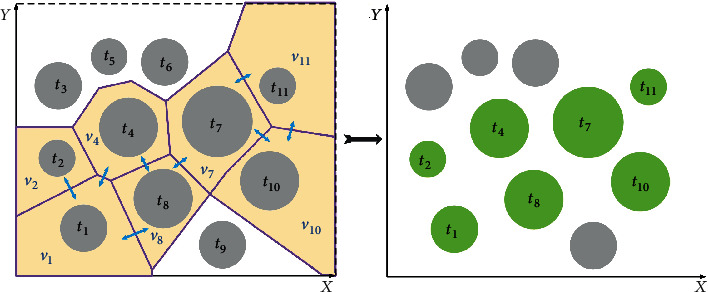
Associated convex polygonal areas and threats.

In summary, as shown in Figure 12, the associated convex polygonal areas set is *V*_associated_ = {*v*_1_, *v*_2_, *v*_4_, *v*_7_, *v*_8_, *v*_10_, *v*_11_}, and the associated threat set is *T*_associated_ = {*t*_1_, *t*_2_, *t*_4_, *t*_7_, *t*_8_, *t*_10_, *t*_11_}.

### 5.3. Search Space Construction

The new spatial search area consists of a closed-loop space connected in sequence by the mission starting point *S*, the boundary docking points, and the mission end point *T*. The key of constructing a new spatial search area are to find the reasonable boundary docking points and determine the spatial search boundary. In order to ensure that the results of the path planning in the new spatial search area remain largely optimal and the computation time of the swarm intelligence algorithm can be reduced. The spatial search area and the boundary docking point should satisfy the following properties.

- The spatial search area is a closed loop area
- The spatial search area contains all associated threats *V*_associated_
- The positions of the boundary docking points should be on the associated threats' borders

To simplify the calculation, according to the properties of the space search boundary, the method for determining the boundary docking points is designed as follows.

- *r*_1_ = *r*_2_

**Case 1 .**

**Figure 13:**
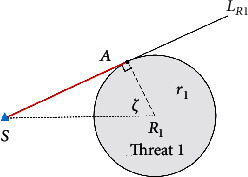
The boundary docking point of the start/end point to the associated threat.

Shown in Figure 13, make the tangent line *L*_*R*1_ of the threat circle 1 through *S* (start or end point), and the tangent point *A* is the boundary docking point of the start/end point to the associated threat, where *AR*_1_⊥*L*_*R*1_, ∠*AR*_1_*S* = *ζ*.

**Case 2 .**

**Figure 14:**
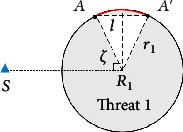
The boundary docking point on the same associated threat.

Shown in Figure 14, cross point *R*_1_ to make the vertical line *l* of *SR*_1_, find the symmetrical point *A*′ of *A* with respect to *l*, and the point *A*′ is the boundary docking point on the associated threat.

**Case 3 .**

Different associated threats have different threat radius. According to the radius of adjacent associated threats, the location of the boundary docking point can be divided into three situations.

**Figure 15:**
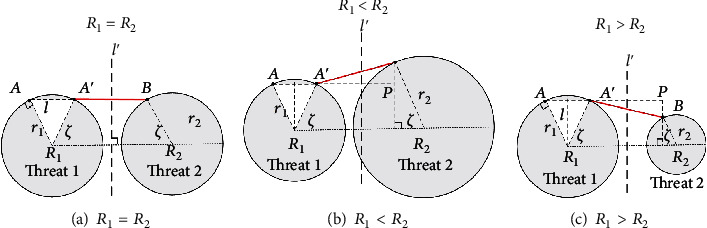
The boundary docking point of different associated threats.

As shown in Figure 15(a), straight line *l*′ is the vertical bisector of line segment *R*_1_*R*_2_, and point *B* is the symmetry point of *A*′ with respect to *l*′. For *r*_1_ = *r*_2_, the point *B* must be on the boundary of threat 2, so point *B* is the boundary docking point of associated threat 1 to associated threat 2.

(ii) *r*_1_ < *r*_2_

As shown in Figure 15(b), straight line *l*′ is the vertical bisector of line segment *R*_1_*R*_2_, and point *P* is the symmetry point of *A*′ with respect to *l*′, where *P* is within the threat 2. Crossing point *P* to make the vertical line *A*′*P* of *R*_1_*R*_2_, and the intersection point *B* with threat 2 is the boundary docking point of associated threat 1 to associated threat 2, where ∠*AR*_1_*R*_2_ = ∠*BR*_2_*R*_1_ = *ζ*.

(iii) *r*_1_ > *r*_2_

As shown in Figure 15(c), straight line *l*′ is the vertical bisector of line segment *R*_1_*R*_2_, and point *P* is the symmetry point of *A*′ with respect to *l*′, where *P* is out of the threat 2. Crossing point *P* to make the vertical line *A*′*P* of *R*_1_*R*_2_, and the intersection point *B* with threat 2 is the boundary docking point of associated threat 1 to associated threat 2, where ∠*AR*_1_*R*_2_ = ∠*BR*_2_*R*_1_ = *ζ*.

**Figure 16:**
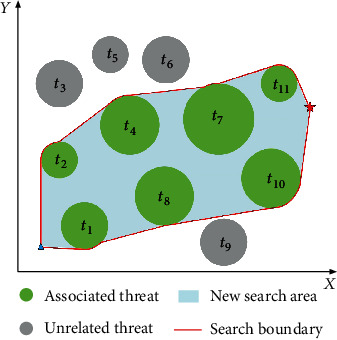
Diagram of new spatial search area.

Due to the location of the associated threats and the mission start/end point were known, the boundary docking point and the search boundary information can be determined by the prior knowledge. The new spatial search area is shown in Figure 16.

## 6. Simulation Experiments

**Figure 17:**
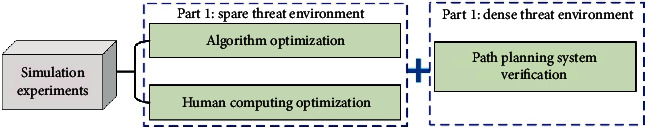
Structure of the simulation.

In this section, for verifying the feasibility and effectiveness of the path planning system based on human-computer hybrid augmented intelligence framework for the UAH, a complete set of simulation experiments are designed. The simulation experiment process is shown in Figure 17.

The simulation experiments are conducted in two-dimension (2D) field. In the 2D field, the size of the UAH flight space is 700km∗400km. The start point is set to [0,0]. The target point is set to [500,0]. We assume that the threats in the planning space are denoted by several circular areas. The related information of the threats is shown in Tables 1 and 2.

### 6.1. Algorithm Optimization Simulation Comparison Results

**Figure 18:**
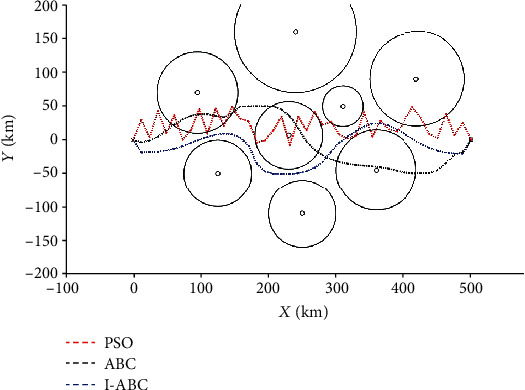
The comparative path planning results.

**Figure 19:**
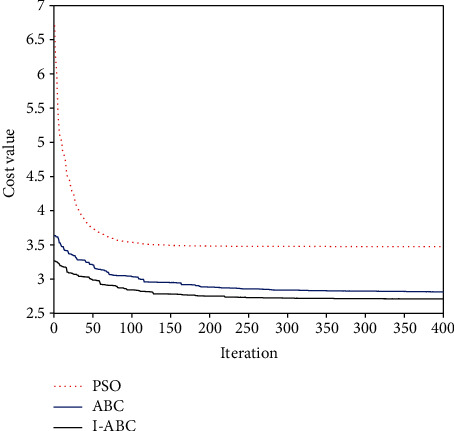
The evolution curves of three algorithms.

**Figure 20:**
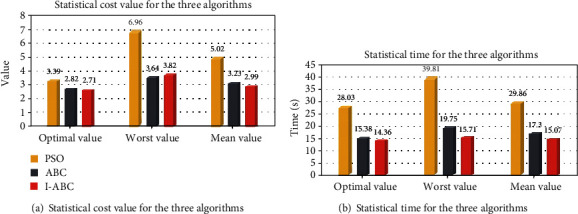
The statistical results for the three algorithms.

To show the superiority of the I-ABC algorithm improved in the algorithm optimization layer, the PSO algorithm, the ABC algorithm, and the I-ABC algorithm are simulated and compared under the same environment model. The maximum iteration number is set as 500, the dimension *D* is set as 40, and the population size *NP* is set as 60. The results are averaged 20 independent runs, and the results of the simulation are illustrated in Figures 18, 19, and 20. The comparison result is listed in Table 3. In this table, the mean, worst, and optimal represent the mean fitness value, the worst fitness value, and the optimal fitness value, respectively.

Figure 18 shows that the intuitive differences of the experimental results between three algorithms in the 2D planning field. We can determine that the result planned by I-ABC algorithm can satisfy the requirements of the UAH path planning. The flight path planned by the PSO algorithm and the standard ABC algorithm have touched and crossed the edge of the threats, and the result of the PSO algorithm is oscillatory.

The convergence curves of the three algorithms are shown in Figure 19. It can be obviously seen from these curves that the convergence effect of the I-ABC algorithm is better than other algorithms. The I-ABC algorithm attains the global optimal value in iteration 221. But the standard ABC algorithm approaches to their optimal value in iteration 278. What is worse is that the PSO algorithm still cannot find its optimal value when the number of iterations is reached.

The intuitive quantitative statistical results are shown in Figure 20 and Table 3. The statistical results of the PSO algorithm are poor, the optimal value is 3.39, the worst value is 6.96, and the average running time is 29.86 s. For the standard ABC algorithm, the optimal value is 2.82, the worst value is 3.64, and the average running time is 17.3 s. Compared with the ABC and PSO algorithms, the planning results of the I-ABC algorithm has smaller cost value and average running time. These denote that the I-ABC algorithm can search for the optimal path stably and also verify the superior performance of the algorithm optimization layer in improving the traditional standard ABC algorithm.

### 6.2. Human Computing Optimization Simulation Comparison Results

**Figure 21:**
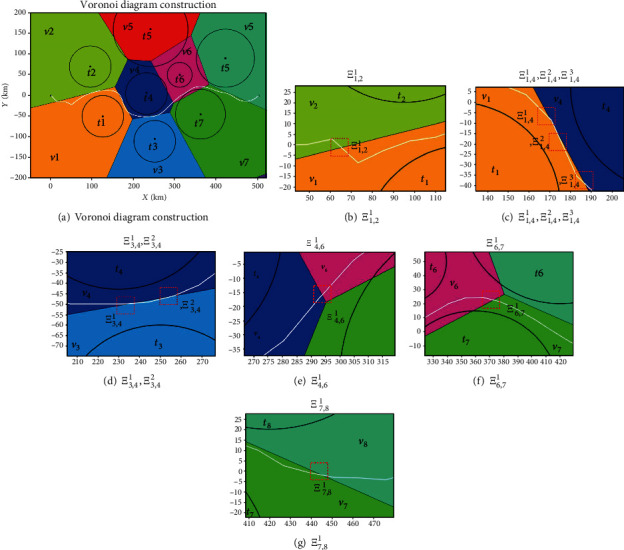
Threat space division.

**Figure 22:**
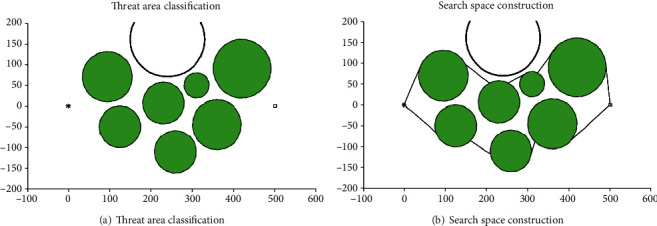
Threat space tailoring.

**Figure 23:**
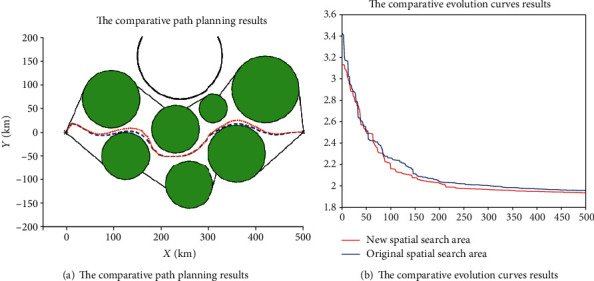
The comparative results for two different spatial search areas.

**Figure 24:**
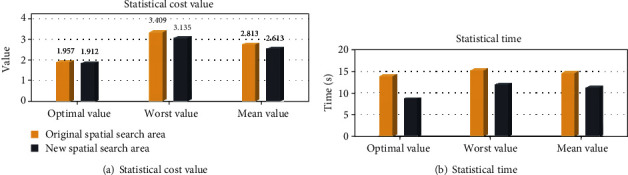
The statistical results of two different spatial search areas.

To further reduce the search time of the flight path and keep its feasibility, the flight space needs to be clipped according to the prior knowledge for constructing a new spatial search space. The process of the space clipping operation is demonstrated in Figures 21 and 22 and Tables 4 and 5. The results are averaged 20 independent runs in the different search spaces, and the results of the simulation are demonstrated in Figures 23 and 24 and Table 6.

Figure 21(a) shows that the original spatial search space was divided into 8 parts include *v*_1_, *v*_2_, ⋯*v*_8_ by Voronoi diagram. According to the intersections of the flight path and the boundary of the convex polygon, the associated threats can be determined. Shown in Figures 21(b)–21(g)) and Table 4, the intersection point set is *Ξ*_1,2_^1^, *Ξ*_1,4_^1^, *Ξ*_1,4_^2^, *Ξ*_1,4_^3^, *Ξ*_3,4_^1^, *Ξ*_3,4_^2^, *Ξ*_4,6_^1^, *Ξ*_6,7_^1^, and *Ξ*_7,8_^1^, the associated convex polygons are *v*_1_, *v*_2_, *v*_3_, *v*_4_, *v*_6_, *v*_7_, and *v*_8_, and the associated threats are *t*_1_, *t*_2_, *t*_3_, *t*_4_, *t*_6_, *t*_7_, and *t*_8_.  Table 5 shows the coordinates of the boundary docking points, by connecting the start/end points with the boundary docking points, we can get the new search space construction as shown in Figure 22.

Figure 23(a) shows the path planning results of the I-ABC algorithm in two different spatial search areas. From the planning results, we can intuitively find that the final results planned by the I-ABC algorithm have almost no differences in two different spatial search areas. The convergence curves of two spatial search areas are illustrated in Figure 23(b). Similarly, from these curves, it can be find that the convergence effect by I-ABC algorithm in the new spatial search areas is slightly better than the original spatial search areas; however, the differences can be almost ignored in practical applications. In other words, the flight path result in the new spatial search area still maintains its optimality in the original spatial search area.

The difference between the flight path of two spatial search areas can be found through the data in Figure 24. For the original spatial search area, the optimal value is 1.957, the worst value is 3.409, and the mean value is 2.813. The optimal search time of the I-ABC algorithm witch runs 20 times is 14.56 s, the worst search time is 15.71 s, and the mean search time is 15.07 s. For the new spatial search area, the optimal value is 1.912, the worst value is 2.778, and the mean value is 2.346. The optimal search time of the I-ABC algorithm witch runs 20 times is 9.05 s, the worst search time is 12.34 s, and the mean search time is 11.69 s. By the comparison, the change in cost value is minimal. And the smoothness and feasibility of the flight path have not changed in the new spatial search area. These denote that the space clipping operation in the human computing layer can indeed reduce the search time of the flight path under the planning result within acceptable changes. Furthermore, the efficiency of the path planning system can be improved.

### 6.3. UAH Path Planning System Simulation Results

**Figure 25:**
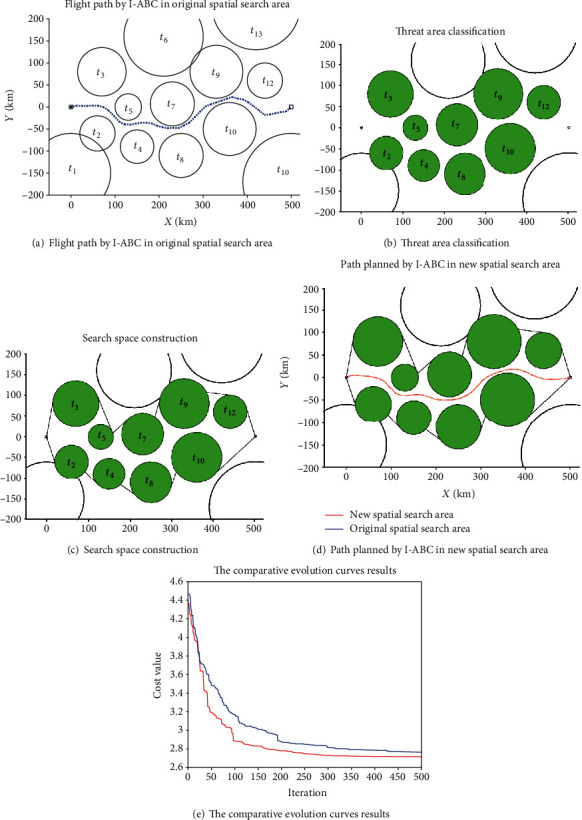
The planning results by I-ABC for the two different spatial search areas.

**Figure 26:**
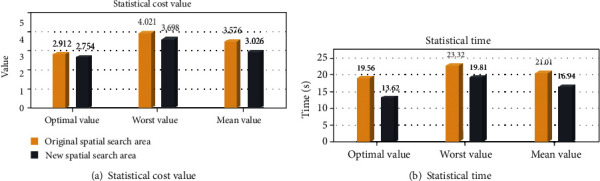
The statistical results of two different spatial search areas.

To further verify the effect of the space clipping operation for the path planning in the complex environment, a complicated environment model with more threats will be used for the simulation. The information of the threats in new environment is shown in Table 2. The maximum iteration number of the I-ABC algorithm is set as 500, the dimension *D* is set as 40, the population size *NP* is set as 60. The simulation results of the path planning system for the UAH in the new map are shown as Figures 25(a)–25(e) and 26 and Table 7.

Figure 25(a) shows that the path planning result by the I-ABC algorithm in the original spatial search area. We can see that the result planned by I-ABC algorithm avoids all threat areas, and it can meet the constraints of the UAH path planning.

Figures 25(b) and 25(c), respectively, show the process of the threat area classification and the search space construction. As shown in Figure 25(b), the associate threats can be identified as *t*_2_, *t*_3_, *t*_4_, *t*_5_, *t*_7_, *t*_8_, *t*_9_, *t*_10_, and *t*_12_. The new spatial search area consists of associated threats and black spatial search boundaries (as shown in Figure 25(c)).

Figure 25(d) shows the path planning results of the I-ABC algorithm in the new spatial search area. Compare with Figure 25(a), the flight path planned in the new spatial search area is similar to the flight path planned in the original spatial search area, both in terms of path smoothness and flight length. The convergence curves of the two spatial search areas are displayed in Figure 25(e). According to these curves, it can be obviously found that the convergence effect by I-ABC algorithm in the new spatial search areas is also similar to the original spatial search areas.

The difference between the planning results of two spatial search areas can be found through the data in Figure 26. For the original spatial search area, the optimal value is 2.912, the worst value is 4.021, and the mean value is 3.576. The optimal search time of the I-ABC algorithm witch runs 20 times is 19.56 s, the worst search time is 23.32 s, and the mean search time is 21.01 s. For the new spatial search area, the optimal value is 2.754, the worst value is 3.698, and the mean value is 3.026. The optimal search time of the I-ABC algorithm witch runs 20 times is 13.62 s, the worst search time is 19.81 s, and the mean search time is 16.94 s. By the comparison, the space clipping operation can maintain the excellent performance of the previous flight path planned by I-ABC algorithm. Moreover, the search time of the path planning system can be further reduced.

## 7. Conclusion

In this paper, a path planning system is presented based on human-computer hybrid augmented intelligence framework for improving the rapidity and optimality of the UAH path planner. At first, the proposed I-ABC algorithm optimizes the follow way of on-looker bees and the update strategy of nectar source. The dynamic evaluation selection strategy and complex optimization method accelerate the convergence rate and maintain the exploration ability of the population. In addition, the space clipping operation based on the attention mechanism reconstructs the spatial search area of the UAH. Unlike adjusting the number of iterations or modifying the parameters of the algorithm, the space clipping operation is directly applied to the optimal solution searched by the I-ABC algorithm. Benefit from this operation, the subsequent search time of the flight path for the path planning system is further reduced. Eventually, the experimental results show that the path planning system based on human-computer hybrid augmented intelligence framework for the UAH can combine the advantages of human and computer for planning a high-quality flight path. This study provides promising results for actual military mission.

## Figures and Tables

**Algorithm 1:**
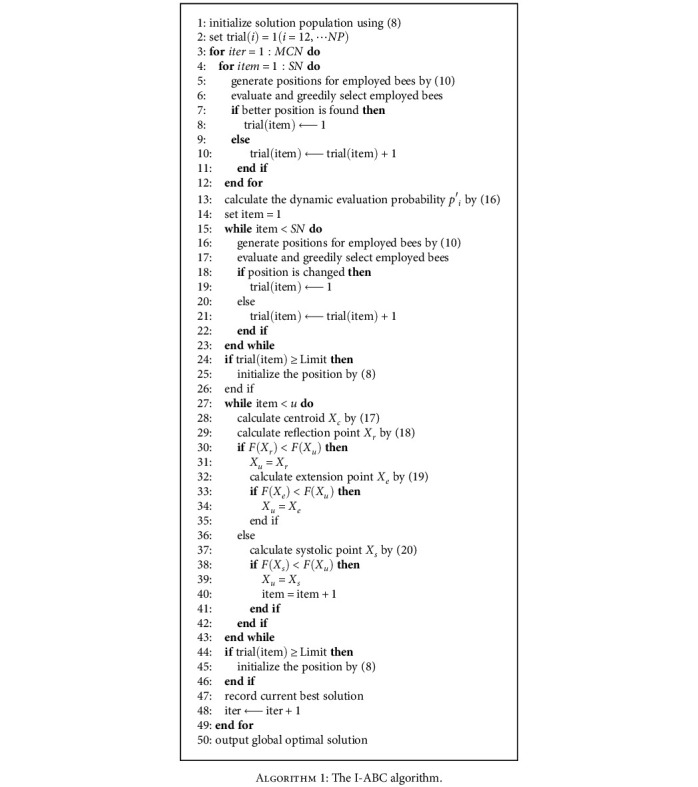
The I-ABC algorithm.

**Table 1:** The information of threat areas.

Threat number	Threat center (km)	Threat radius (km)
*t*_1_	[125,-50]	50
*t*_2_	[95,70]	60
*t*_3_	[250,-110]	50
*t*_4_	[230,7]	50
*t*_5_	[240,160]	90
*t*_6_	[310,50]	30
*t*_7_	[360,-45]	60
*t*_8_	[420,90]	70

**Table 2:** The information of threat areas.

Threat number	Threat center (km)	Threat radius (km)
*t*_1_	[0,-150]	90
*t*_2_	[60,-60]	40
*t*_3_	[70,80]	55
*t*_4_	[150,-90]	38
*t*_5_	[130,0]	30
*t*_6_	[210,160]	90
*t*_7_	[230,7]	50
*t*_8_	[250,-110]	50
*t*_9_	[330,80]	60
*t*_10_	[360,-50]	60
*t*_11_	[500,-170]	110
*t*_12_	[440,60]	40
*t*_13_	[420,230]	100

**Table 3:** Results comparison for three algorithms.

Algorithms	PSO		ABC		I-ABC	
Performances	Value	Time(s)	Value	Time(s)	Value	Time(s)
Optimal	3.39	28.03	2.82	15.38	2.71	14.36
Worst	6.96	39.81	3.64	19.75	3.28	15.71
Mean	5.02	29.86	3.23	17.3	2.99	15.07

**Table 4:** Associated convex polygons and threats.

Intersection points	Associated convex polygons	Associated threats
*Ξ*_1,2_^1^	*v*_1_, *v*_2_	*t*_1_, *t*_2_
*Ξ*_1,4_^1^	*v*_1_, *v*_4_	*t*_1_, *t*_4_
*Ξ*_1,4_^2^	*v*_1_, *v*_4_	*t*_1_, *t*_4_
*Ξ*_1,4_^3^	*v*_1_, *v*_4_	*t*_1_, *t*_4_
*Ξ*_3,4_^1^	*v*_3_, *v*_4_	*t*_3_, *t*_4_
*Ξ*_3,4_^2^	*v*_3_, *v*_4_	*t*_3_, *t*_4_
*Ξ*_4,6_^1^	*v*_4_, *v*_6_	*t*_4_, *t*_6_
*Ξ*_6,7_^1^	*v*_6_, *v*_7_	*t*_6_, *t*_7_
*Ξ*_7,8_^1^	*v*_7_, *v*_8_	*t*_7_, *t*_8_

**Table 5:** Boundary docking points.

Associated threats	Boundary docking points	Distribution coordinates (km)
*t*_1_	*A*	[77.45, −65.45]
	*A*′	[160.4, −85.36]
*t*_2_	*B*	[41.54,97.24]
	*B*′	[148.5,97.24]
*t*_3_	*C*	[209.6, −117.8]
	*C*′	[308.4, −117.8]
*t*_4_	*D*	[207.3,51.55]
	*D*′	[252.7,51.55]
*t*_6_	*E*	[296.4,76.73]
	*E*′	[323.6,76.73]
*t*_7_	*F*	[317.6, −87.43]
	*F*′	[402.4, −87.43]
*t*_8_	*G*	[370.5,139.5]
	*G*′	[489,101]

**Table 6:** Results comparison for two spatial search areas.

	Original spatial search area		New spatial search area	
Performances	Cost value	Time (s)	Cost value	Time (s)
Optimal	1.957	14.36	1.912	9.05
Worst	3.409	15.71	3.135	12.34
Mean	2.813	15.07	2.613	11.69

**Table 7:** Results comparison for two spatial search areas.

	Original spatial search area		New spatial search area	
Performances	Cost value	Time (s)	Cost value	Time (s)
Optimal	2.912	19.56	2.754	13.6
Worst	4.021	23.3	3.698	19.81
Mean	3.576	21.01	3.026	16.94

## Data Availability

The experimental data of this study are included within the article.
